# Enhancing employee experience and workplace well-being in oncology: a bottom-up co-construction approach in healthcare institutions in Québec

**DOI:** 10.1186/s12913-026-14881-9

**Published:** 2026-06-05

**Authors:** Labante Outcha Dare, Roxane Borgès Da Silva, Alain Marchand, Kathleen Bentein, Mélanie Lavoie-Tremblay, Christine Maheu, Marie-Andrée Fortin, Sara Soldera, Alexandre Prud’Homme, Carl Ardy Dubois

**Affiliations:** 1 CHU Sainte-Justine, Centre hospitalier mère-enfant, Direction de la qualité, évaluation, performance et éthique, Bureau 403, 5757 Decelles, Montréal, Québec H3S 2C3 Canada; 2 École de santé publique de l’Université de Montréal (ESPUM), Département de gestion, d’évaluation et de politique de santé (DGEPS), 7101, avenue du Parc, 3e étage, bureau 3014-8, Montréal, Québec H3N 1X9 Canada; 3 École de relations industrielles de l’Université de Montréal. Pavillon Lionel-Groulx C. P, succursale Centre-ville Montréal (Québec), 7101, avenue du Parc, 3e étage, bureau 3014-8, Montréal (Québec), H3N 1X9 Canada; 4 ESG Université du Québec à Montréal, Département d’organisation et ressources humaines, C.P. 8888, Succursale Centre-ville, Montréal, Québec H3C 3P8 Canada; 5 Faculté des sciences infirmières de l’Université de Montréal. Pavillon Marguerite-d’Youville, 2375, chemin de la Côte-Sainte-Catherine, Montréal, Québec H3T 1A8 Canada; 6 Centre de recherche de l’Institut universitaire en santé mentale de Montréal, 7331 rue Hochelaga, Montréal, Québec H1N 3V2 Canada; 7 École des sciences infirmières Ingram de l’Université McGill, 680 Sherbrooke West, Suite 1800, Montréal, Québec H3A 2M7 Canada; 8 Institut de recherche du Centre universitaire de santé McGill, 1650 Avenue Cedar, Montreal, Québec H3G 1A3 Canada; 9 Centre intégré de cancérologie de Laval (CISSS de Laval), 1755 Bd René-Laennec, Laval, Québec H7M 3L9 Canada; 10 Faculté de médecine de l’Université de Montréal, Département de radiologie, radio-oncologie et médecine nucléaire. Pavillon Roger-Gaudry, porte S-716, C.P. 6128, Succursale Centre-ville, Montréal, Québec H3C 3J7 Canada; 11 Centre universitaire de santé McGill (CUSM), Centre de cancérologie Cedars, 1001 boulevard Decarie, Montréal, Québec H4A 3J1 Canada; 12 Faculté de médecine et des sciences de la santé, Département d’oncologie Gerald Bronfman, Université McGill, 3001, 12e avenue Nord, Sherbrooke, Québec J1H 5N4 Canada; 13 Centre de recherche en santé publique (CReSP), 7101, avenue du Parc, Montréal, Québec H3N 1X9 Canada; 14 Centre interuniversitaire de recherche en analyse des organisations (CIRANO), 1130, Sherbrooke Ouest, suite 1400, Montréal, Québec H3A 2M8 Canada

**Keywords:** Oncology, Environment, Co-construction, Bottom-up, Participatory approach, Continuous improvement, Contextual factors.

## Abstract

**Background:**

The commitment of health personnel in initiatives for improving their working conditions is recognised as a key condition for the success and the sustainability of such initiatives. Healthcare organisations are faced with the challenge of deploying strategies needed to mobilise this commitment. The objective of this article is to present the results of a qualitative evaluation of a bottom-up co-construction project aimed at engaging oncology staff in four Québec healthcare organisations in a process of transformation and improvement of their working environment.

**Methods:**

As part of our constructivist approach, we utilised a qualitative method, which involved conducting one-on-one interviews and gathering documentary data, including survey results, to assess the development and implementation of an intervention across four oncology units. We conducted one-on-one interviews from January 26, 2023 to March 5, 2023 with 17 workers from different categories. We collected documentary data that cover the pre-implementation activities, the co-construction workshops, and the intervention implementation. All collected data were coded and analysed using QDA Miner 6.0, and our findings were validated iteratively throughout the project, involving regular interaction with participants.

**Results:**

Fourteen areas of vulnerability emerged across the four dimensions studied, and six were targeted by workers as priorities: emotional exhaustion; role conflict; ability to learn; leadership; team cohesion; and communication. Interventions developed to address the prioritised areas included: co-development workshops; training sessions aimed at enhancing workers’ control over their working environment; team connectivity through professional and social activities; staffing and workload reviews; support from a psychosocial professional; and coaching. According to workers, most improvements occurred in two targeted areas, team cohesion and communication, even in the units where these issues were not prioritised at first. Participants pinpointed some factors that facilitated the implementation of the intervention and its impact (engagement, organisational support, the bottom-up approach) and others that created constraints (staff shortages, conflicting priorities, level of commitment).

**Conclusions:**

This exploratory work offers insightful perspectives on how a bottom-up co-construction approach can serve as a lever to engage workers to improve their work experience. It may inspire other healthcare organisations, both in oncology and in other fields of activity.

**Supplementary Information:**

The online version contains supplementary material available at 10.1186/s12913-026-14881-9.

## Background

Oncology care unfolds within a uniquely demanding work environment, where professionals are exposed daily to high emotional loads and clinical complexity. The nature of cancer care—marked by frequent exposure to suffering, end-of-life situations, and complex therapeutic decisions—places significant psychological and emotional demands on healthcare workers. These challenges are further intensified by the need for continuous adaptation to evolving medical practices and technologies [1–3]. Oncology staff often navigate situations where curative options are limited. Yet, they are expected to communicate difficult yet honest information to patients with sensitivity, even when invasive treatments offer only marginal benefits to the patient’s quality of life. This dynamic frequently generates emotionally intense situations, which can lead to anxiety, moral distress, burnout, and a profound sense of helplessness [4–6].

Several interrelated factors contribute to making oncology a high-stress professional context. First, the emotional burden associated with close interactions with patients facing terminal illness and their families is substantial. Second, systemic issues such as resource constraints, ethical dilemmas, and organisational expectations add to the complexity of care delivery. Professionals often have the responsibility to communicate difficult information and pursue aggressive treatments, which leads to internal conflict and moral distress [7]. Additionally, oncology workers often suppress their own emotional reactions, neglecting self-care to maintain professional composure [8]. Over time, this suppression can evolve into psychological exhaustion and disengagement, especially when workplace conditions fail to support emotional resilience.

The consequences of such persistent stress are wide-ranging and detrimental. Burnout—characterised by emotional exhaustion, depersonalisation, and reduced personal accomplishment—is common in oncology settings and significantly compromises both caregiver well-being and patient outcomes [9]. Studies report associations between burnout and decreased job satisfaction, high absenteeism, increased turnover, and diminished quality of care [10]. Clinically, burnout can manifest as fatigue, insomnia, somatic complaints, irritability, and even depression [11]. Left unaddressed, it can escalate to compassion fatigue, interpersonal conflicts, and, ultimately, disengagement from the profession [4, 12–14]. Recent evidence of a meta-analysis [15] indicates that the average proportions of emotional exhaustion, depersonalisation, and reduced personal accomplishment were 32%, 21%, and 26% among oncology nurses and 32%, 26%, and 25% among medical staff.

Rethinking the work environment becomes essential. To this end, improving the professional experience of oncology workers requires not only individual coping strategies, but also systemic interventions that support team resilience. One promising avenue lies in the adoption of a continuous improvement approach—an organisational strategy that promotes shared responsibility, frontline engagement, and incremental yet meaningful change. This continuous improvement is defined as a cultural shift towards a new philosophy of becoming a self-analytical, self-critical learning organisation that enables frontline staff to identify the root causes of problems in systems and processes and develop solutions leading to small incremental changes that, when combined, produce significant results [16, 17]. Far from relying on top-down directives, this model empowers staff to collaboratively identify barriers and co-create practical solutions that enhance both human relations and the care environment.

Responding to prior findings that highlighted the perception of oncology units as unstable and poorly supportive work environments [14, 18], a Canadian research team implemented a bottom-up, co-constructed intervention in four oncology units in Québec. This article presents the qualitative evaluation of this intervention, focussing on the development, implementation, and perceived effects. The aim is to show how the co-construction process, through collaborative strategies, engages staff in a process of collaboration, transformation, and improvement of their working environment.

## Methods

**Fig. 1:**
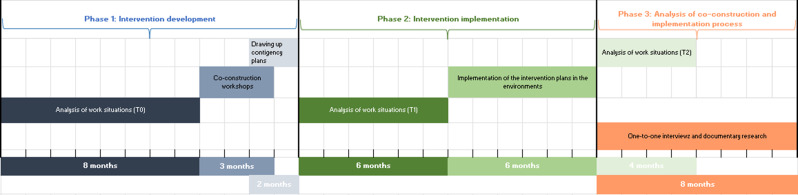
Operational framework and chronology of the project stages

The study involved cancer care teams in four institutions located in the greater Montreal region. One Integrated Health and Social Services Centre was identified as **Unit A**, two Integrated University Health and Social Services Centres were identified as **Unit B** and **Unit D**, and one university health centre was identified as **Unit C**. The study was operationalised following three steps (Fig. 1) over a period of 32 months.

### Phase 1: Intervention development

The first phase aimed to develop a tailored intervention plan that considered the specific contexts of each of the four participating cancer units. During this first phase of the intervention, two activities were conducted. The first activity was a detailed analysis of work situations in the four units, based on a staff survey. This baseline survey used a series of tools covering four dimensions and 19 potential domains of vulnerability:

- Three related to resources at work: supervisor support, peer support, and decision latitude [19];
- Six related to work demands: psychological demand and emotional demand [20]; work-family balance [21]; role conflict and role ambiguity [20]; and burnout [22];
- Five related to team resilience: ability to respond, ability to monitor, ability to anticipate, ability to react, and ability to learn [23, 24]; and,
- Five related to team effectiveness: leadership, communication, common understanding, conflict management, and cohesion [25, 26].

The second activity was a series of four co-construction workshops, each lasting approximately 90 min, in which the cancer teams in each unit aimed to produce three deliverables: identification of main areas of vulnerability in their work experience, determination of those areas of vulnerability that need priority attention, and development of an intervention plan to address those prioritised areas. The first workshop addressed the two first deliverables. These team workshops, developed based on a previously established research protocol that included, among other elements, the working components, were facilitated by the research project coordinator in collaboration with the unit team leaders in each unit. The interprofessional composition of the teams was defined by research project coordinator in collaboration with the unit managers.

The results of the work situation survey were presented to the participants and provided the information needed to identify areas of vulnerability and designate priorities. The second workshop provided an opportunity for the participants to discuss a range of strategies that would address the previously prioritised issues. The third workshop involved making a choice among the strategies discussed previously, based on their relevance and feasibility. The fourth workshop’s objective was to define specific actions to be taken to implement the strategies retained and integrate them in an intervention plan. The plans were developed from February to July 2022.

### Phase 2: Intervention implementation

The implementation was carried out through the dissemination of intervention plans within each unit, the establishment of a steering structure in each unit with the appointment of a resource person, the development of monitoring tools, and the organisation of regular meetings with the resource persons in each unit. It should be noted that each implementation team included members of the project steering committee. The actual implementation of intervention plans began in August 2022 and was completed in February 2023.

The present article focuses exclusively on the qualitative component of the project. It does not report the quantitative results arising from the data collection activity conducted during the analysis of work situations at T1, as illustrated in Fig. 1. These quantitative findings may be the subject of a separate future publication, notably through the analysis of the pre-implementation, implementation and post-implementation questionnaires administered in each of the units studied [19, 20, 23, 26–28].

### Phase 3: Analysis of co-construction and implementation process

A qualitative analysis was conducted to assess the co-construction approach, the implementation process of the intervention plans, and their resulting effects. Two complementary methods were employed: a documentary review and 17 structured one-on-one interviews using an interview guide that we developed as part of this study (Supplementary material 1: Interview guide).

The documentary analysis, carried out in April 2023, used materials compiled during the diverse phases of development and implementation of the intervention plans. Concurrently, interviews were carried out between January 26 and March 5, 2023. In collaboration with the heads of each oncology unit, a list of health workers from different categories was established and email invitations were sent to all targeted workers. The selection of workers for the list of participants was carried out in a considered manner, considering the research project coordinator’s experience and knowledge of the field. The semi-structured interviews, which lasted between 20 and 76 min, covered a range of dimensions related to the intervention, including participants’ perceptions regarding its nature, intended goals, implementation stages, relevance, and effectiveness. Interviews also explored challenges encountered during the intervention and perceptions of its strategic context within their respective units.

We handled all collected data confidentially. Interview transcripts were first anonymised to ensure participant privacy, then systematically coded according to emerging themes. The data analysis, conducted using QDA Miner 6.0, began with a close reading of the transcripts to gain familiarity with the content. All coding was performed by the same individual who conducted the interviews and compiled the documentary review. Specifically, the coding framework was developed through an iterative process and continuously refined in discussion with the research team, allowing for regular validation of emerging themes and interpretation consistency. In addition, coding decisions were systematically reviewed and discussed within the team to ensure coherence and minimise subjective bias. The resulting codes (Supplementary material 2: Code tree) were grouped into categories aligned with the study’s core research questions.

## Results

### Intervention development

The initial phase provided an opportunity for workers to provide their assessments of their work situations, identify areas of vulnerability in their work experience with guidance from the research team, determine prioritised vulnerability areas that must be addressed, and find solutions for those prioritised areas. Preparatory work carried out included a kickoff meeting, the creation of the participant list for the survey and the selection of participants for the co-construction workshops based on a purposive sampling, the selection of employee ambassadors and a resource person within each unit, and the creation of an online forum for each unit.

### Situation analysis and identification of key areas of vulnerability

In three units, over 50% of workers participated in the survey (ranging from 53% to 80%), while only 31% in the fourth unit participated. A total of 256 individuals, covering six distinct occupational groups, participated, spanning a variety of professional roles (Table 1). **Unit A** had the largest number of participants (*n* = 129), followed by **Unit B** (*n* = 61), **Unit C** (*n* = 46), and **Unit D**, which had the smallest sample (*n* = 20).

**Table 1:** Participant profile in the situation analysis phase

Healthcare organisation	Medical staff	Nursing staff	Paratechnical personnel, auxiliary services, and trades	Technicians	Professionals	Administrative staff	Total
**Unit A**	23	32	10	27	21	16	129/206 (63%)
**Unit B**	21	23	3	0	7	7	61/197 (31%)
**Unit C**	0	35	11	0	0	0	46/87 (53%)
**Unit D**	2	9	2	0	2	5	20/25 (80%)
**Total**	46	99	26	27	30	28	256

Source: Research document and documentary synthesis of the intervention

With guidance from the research team, the first of a series of four workshops was used to determine key areas of vulnerability based on the four dimensions. Overall, for the four units, fourteen areas of vulnerability were identified as shown in Table 2: decision-making latitude and instrumental support (resources); psychological and emotional requirements, emotional exhaustion, role conflict, and work-family balance (demands); ability to anticipate, ability to monitor, ability to respond, and ability to learn (resilience); communication and coordination, leadership, and cohesion (team effectiveness). Three areas were identified for **Unit D**, five for **Unit A**, six for **Unit C**, and seven for **Unit B**. Emotional exhaustion was identified as the most prevalent issue, present across all four units. Communication was identified as an issue in three units, and decision-making latitude was a concern in two units (Table 2).

**Table 2:** Situations in units at T0 – participants and targeted areas

	Unit (Areas of vulnerability per unit)			
	A	B	C	D
Dimension	*Sub-dimensions*			
**Resources**	*n.i.*	*n.i.*	Decision-making latitude	Decision-making latitude
**Demands**	Psychological demand Emotional demand Emotional exhaustion Role conflict	Psychological demand Emotional demand Emotional exhaustion	Work-family balance Emotional exhaustion	Emotional exhaustion
**Resilience**	*n.i.*	Ability to anticipate Ability to monitor Ability to respond Ability to learn	*n.i.*	*n.i.*
**Team effectiveness**	Communication	*n.i.*	Leadership Communication Cohesion	Communication

n.i.: No elements of the sub-dimension identified by participants

### Workers’ prioritisation of identified areas of vulnerability and search for solutions

Following the identification of the main areas of vulnerability, the series of four workshops held in each of the units was used to decide the prioritisation of these areas and search for solutions. Each of the four co-construction workshops mobilised a core group of workers composed of various job groups and sectors of activity (with a maximum set at 15). On average, nine workers participated in each workshop in **Unit A**, ten in **Unit B**, nine in **Unit C**, and four in **Unit D**.

As shown in Table 3 below, two issues were prioritised in three units (**A**, **B**, and **D**) while the remaining unit prioritised three (**C**). Emotional exhaustion was consistently identified as a priority across all four units. In **Unit A**, role conflict was identified as a second priority, while in **Unit B** and **D**, the second priority was ability of learning and communication respectively. In **Unit C**, the two other priorities were team leadership and team cohesion (Table 3). To address these prioritised elements, workers, by unit, developed an action plan that included diverse types of activities:

- Co-development workshops and coaching for management teams,
- Training sessions aiming at enhancing workers’ control over their working environment,
- Brainstorm sessions with teams on selected issues,
- Feedback sessions on difficult situations experienced,
- Intensification of team connectivity opportunities through professional and social activities (regular team meetings, community of practice, highlights of achievements, birthday greetings, remission celebrations with patients),
- Review of work organisation—staffing and workload reviews, support from a psychosocial professional.

### Intervention implementation

The intervention was implemented over a period of 6–8 months in each facility. During the implementation stage, a key contact for each cancer unit was identified (the team leader or a senior manager) and tasked with ensuring the smooth running of activities in the intervention plan within the allotted period. This person was supported by a project manager and a research assistant deployed by the research team. Support was also provided by the two principal investigators. External consultants were mobilised for some activities, such as training on specific topics. Systematic monitoring was ensured with a monthly meeting involving the contact person in the unit, the project manager, and the research assistant. Throughout implementation, various communication activities were carried out to promote the project within the facility, inform staff, and stimulate workers’ participation. Two of the four oncology units (**Unit A** and **Unit C**) used financial incentives to encourage worker attendance in certain activities (e.g., workshops, training sessions). Managers were flexible in accommodating workers’ participation in project activities. As shown in Table 3 above, across all units studied, most of the activities planned in each of the four units were implemented, except for **Unit C**.

**Table 3:** Summary of prioritised issues, planned activities, and implementation activities

Unit	Issue Prioritised	Planned activity	Status
**A**	Role conflict	• Two working sessions with administrative staff	Completed
		• Two co-development workshops with management committee (psychological safety, recognition)	Completed
	Emotional exhaustion	• Three lunchtime training sessions (letting go of the uncontrollable, verbalising dissatisfaction, work-life balance)	Completed – Over 14 sessions
		• At least four multidisciplinary meetings on difficult cases	Completed – Once
		• Co-development workshop on psychological safety and success	Completed
		• Recognition activities and best practices identified ✓ Find best practices and development needs ✓ Co-development workshop for management committee (psychological safety, recognition) and dissemination of successes ✓ Personalised support to management committee of the Interdisciplinary Local Design Committee	Completed
**B**	Resilience (learning)	• Four-modules training on integrated patient pathway including palliative care and bereavement	Completed – Over nine sessions
		• Expert workshop to promote a learning culture that encourages sharing mistakes without judgment	Completed
	Emotional exhaustion	• Two informal meeting points to promote team spirit	Completed – Over seven meetings
		• Community of practice meeting to share best practices	Completed
**C**	Leadership	• Five-minute end-of-meeting updates for nursing assistants	Not completed
		• Communication notebook reassessment and systematisation, if needed	Not completed
		• Nurses answering calls when nursing assistants busy	Completed
		• Coaching for assistant head nurses	Not completed
	Team cohesion	• Three multidisciplinary training sessions (letting go of the uncontrollable, verbalising dissatisfaction, and establishing healthy communication)	Completed – Over two sessions
		• Highlight social events ✓ Monthly birthday celebration ✓ Thursday celebrations with patients, cake, and music	Not completed
		• Orientation period restructuring and integration	Not completed
		• Strengthen the preceptorship system: pairing each junior nurse with one or two senior nurses	Incomplete
		• Employee survey on development needs	Completed
		• Increase nursing assistants ratio	Completed
		• Increase recruitment	
		✓ Social media campaign development	Completed
		✓ Communicate critical care bonus updates	Not completed
		• Restructuring nurse/assistant tasks	
		✓ Autonomous workload sharing for nurses’ difficult cases	Completed
		✓ Task model selection for nursing assistants	Completed
	Emotional exhaustion	• Training sessions and on-site support provided to team members by a psychosocial professional	Completed – Over five sessions
		• Remission celebrations	Not completed
		• Thank-you notes from patients and families displayed	Completed
**D**	Communication	• Three preparatory meetings for multidisciplinary meetings with cross-participation	Completed
		• Team assessment with interviews to target dysfunctions	Completed
		• Two workshops on team cooperation and building a renewed team culture through healthy direct communication	Completed – Over four sessions
	Emotional exhaustion	• Tailored coaching support for manager on crisis management and plan implementation	Completed

In **Unit A**, two issues were prioritised: role conflict and emotional exhaustion.

To address role conflict, two activities were completed as planned. First, two working sessions were organised with administrative staff to identify role conflicts and propose solutions. Second, two co-development workshops were held with members of the management committee, focussing respectively on psychological safety and recognition.

To address emotional exhaustion, four activities were carried out with the intent to raise awareness of sources of stress, provide opportunities for open dialogue, and empower both managers and their team members to gain control over their work environment. First, three training modules were developed and offered during lunch breaks over 14 sessions to accommodate the largest number of participants, covering topics such as letting go of the uncontrollable, expressing dissatisfaction, and achieving work-life balance. Second, during service meetings a period was reserved for discussion on difficult situations to encourage peer support. Third, a best practices workshop was organised, and coaching was provided to the management team to promote employee recognition activities. Fourth, a co-development workshop on psychological safety and success was conducted for the members of the management committee.

In **Unit B**, two issues were prioritised: ability to learn and emotional exhaustion.

To address the first issue, two activities were deployed. Four training modules on sensitive themes including palliative care, palliative sedation, medical assistance in dying, and end-of-life support were developed and offered over nine training sessions. In addition, an expert-led workshop was held on psychological safety, with the intent of fostering a supportive team culture where errors could be shared without judgment.

To address emotional exhaustion, this unit focussed on increasing occasions of interaction between team members, with the intent to create a collective learning dynamic and a strong sense of community among employees. A series of seven team meetings were organised, creating spaces for open discussion on challenging issues, including the introduction of new medications (e.g., a new COVID-19 drug), unit audit results, elder abuse reporting, and teaching drug infusion techniques. Other meetings were planned to share best practices but were not held on a regular basis.

In **Unit C**, three issues were prioritised: leadership, team cohesion, and emotional exhaustion.

To address leadership, some activities were initially planned to reinforce leadership by fostering clearer communication and better team coordination but not fully completed. While nursing assistants were expected to receive updates at the end of meetings and a communication notebook was to be reassessed and systematised, these plans were not implemented. Nonetheless, nurses did respond directly to calls when nursing assistants were unavailable. Coaching for assistant head nurses was planned but not completed.

To improve team cohesion, several activities were launched. Three multidisciplinary training sessions over two sessions were completed on letting go of the uncontrollable, expressing dissatisfaction, and direct communication. Social events such as monthly birthday celebrations and Thursday gatherings were planned though not carried out. Orientation and integration of new staff were redesigned but not implemented, and the preceptorship system (pairing junior nurses with senior staff) was only partially applied. Staff development needs were surveyed, and measures were taken to keep teams informed. Since nurse/assistant involvement in our project was restricted, the expected intensity of the activities was difficult to attain in some cases (e.g., autonomous workload sharing for nurses’ difficult cases).

To address emotional exhaustion, actions included ensuring a psychosocial professional’s presence on-site at regular times through a series of five sessions. Celebrations of remissions were planned but not achieved, whereas thank-you notes from patients and families were displayed in staff areas as a form of recognition.

In **Unit D**, two issues were prioritised: communication and emotional exhaustion.

To improve communication, three preparatory meetings were organised to launch multidisciplinary meetings, with cross-participation between oncology and hematology teams. A team assessment was carried out with individual interviews to identify dysfunctions. In addition, two workshops were developed and delivered to strengthen direct collaboration and build a renewed team culture, addressing healthy communication and protection from emotional burdens.

To reduce emotional exhaustion, tailored coaching support was provided to the manager to enhance their crisis management skills with the intent to strengthen their ability to prevent these situations in the team and create conditions for a healthier work environment.

### Analysis of co-construction and implementation process

One of the key outcomes that emerged from the analysis was the engagement process facilitated by this project. The opportunities for worker engagement included: responding to different rounds of surveys, bringing their input to the identification and prioritisation of main areas of vulnerability in their work environment, contributing to action plans within the construction workshops, connecting more regularly with other team members, and implementing the action plans.

*It was the same design process*,* which consisted of four workshops… and once the workshops were completed*,* each unit had its own action plan… At the implementation stage*,* there were meetings with the unit managers*,* presentation of the action plan… After that*,* there were monthly meetings with the managers of each unit to review progress and identify any issues. The action plans were implemented over a period of approximately six months in each organisation…***(Interviewee 17)**

*There is a stage where we reflect on how to implement the project within the care unit. There was a stage where we involved people to help us with this reflection. Then we held meetings to identify the issues to be addressed. Afterwards*,* we moved to a stage where we sought solutions*,* and the final stage was implementing those solutions. We will then measure the impact of the selected solutions and interventions…***(Interviewee 14)**

Despite the high workload inherent in oncology and the additional strain caused by the pandemic, participants expressed their appreciation for the opportunities they were given to reflect on their working conditions.

*Uh… yes*,* I would say… because*,* well*,* we haven’t finished it yet*,* as I consider this to be something that will always be ongoing… But I can see progress*,* I can see improvements… We are seeing improvement. We can see that we are on the right track*,* but we need to continue in the same direction. ***(Interviewee 06)**

Seventy-one percent of the 43 participants in the co-construction workshops (Unit A = 14, Unit B = 7, Unit C = 19, Unit D = 3) indicated that the process met their expectations and expressed a high level of satisfaction, with an average rating of 4 out of 5 in response to the two questions they were asked: *“Did the workshop meet your expectations?”* and *“How would you rate the overall conduct of the workshop (5 being the highest)?“*. From the perspective of one-to-one interviewees, the approach taken in this project was inclusive, perceived as both a learning process and an innovative co-construction initiative allowing them to reflect on their practices and work environments while seeking to develop the most appropriate solutions by themselves, for themselves.

According to the participants, despite considerable challenges, most of the solutions developed to address the identified priority areas of vulnerability within each unit were implemented. Although the project did not result in a radical transformation of their work environment—particularly regarding perceived burnout—it was nonetheless associated with several improvements in their work experience. The reported benefits included strengthened team cohesion, improved communication, increased attentiveness among colleagues, a reduction in workplace tensions, and enhanced individual and collective capacities to navigate complex frontline challenges. For managerial staff, the process also served as an opportunity for reflective learning and deepened awareness of team dynamics and frontline realities (Table 4).

**Table 4:** Examples of quotations illustrating the reported improvements by interviewees

Quotations	Interviewee
*“Well*,* yes*,* as I was saying*,* multiple meetings*,* case discussions*,* and educational components within meetings that are no longer exclusive to certain professions but are now more inclusive.”*	**03**
*… I know that this is something that has been implemented*,* in fact*,* to improve communication within our team… It is a newsletter that has been put in place*,* which is sent to the team every week to keep everyone informed about what is happening on the ward*,* any changes*,* upcoming matters*,* and issues to be addressed together.”*	**05**
*“When the team is better equipped*,* when the team pulls together*,* when the team is able to move forward using the tools that have been put in place… it has fostered a spirit of collaboration among them…"*	**06**
*“Well*,* I would say that what we have really put in place is the sharing of training and knowledge. For example*,* I have an employee who attended a conference in Toronto on apheresis over a weekend. He then used the hour usually allocated for our meeting*,* upon his return*,* to share with the team all the new information on apheresis…"*	**07**
*“The effects? Well*,* basically*,* I can see that it has led to a certain consolidation and strengthened bonds within the team. Especially with the fact that we can do more group activities*,* I can see a certain cohesion developing among team members.”*	**08**
*“… it was positive because we communicated and talked a bit more. When we had a meeting*,* we had a plan*,* we looked ahead*,* and that was positive.”*	**10**
*“… it seems to me that people are more sensitive to others*,* and they pay more attention to others*,* to people’s reactions*,* to the way they speak; they are more respectful and in better spirits.”*	**11**
*“[For] me*,* people feel a strong team spirit. People feel collegiality. People feel that they are comfortable asking questions. New recruits feel supported. We are also working on a specific orientation programme that will also help with clinical issues and clinical questions…"*	**14**
*“… I thought it was a great opportunity to find ways to support*,* help*,* and equip the teams. […] I think what was most beneficial was the training provided to the teams.”*	**15**

Some specific benefits have been highlighted for each unit. **Unit A** participants emphasised the previously underappreciated significance of administrative agents, indicating a shift in recognition of their essential role within the organisational ecosystem. This suggests that the intervention facilitated a re-evaluation of internal roles and fostered greater appreciation of support functions. In **Unit B**, participants emphasised improvements in team interactions with more spontaneous meetings and normalisation of constructive discussions about mistakes leading to some adjustments in management practices. It was reported that the action plan resulted in an increase in peer attentiveness, more informal knowledge-sharing, and improved post-training interactions, suggesting a more positive work climate overall. In **Unit C**, participants emphasised the contribution of the project in terms of team building, collaboration, and managers’ awareness of the work environment. It was reported that the action plan provided opportunities for personal development among managers, fostered team cohesion, encouraged more effective communication, provided quick access to relevant information, and resulted in a renewed collaborative spirit and a stronger sense of being heard and valued at work. A heightened vigilance towards early signs of dissatisfaction was observed. In **Unit D**, participants also emphasised improvement in team dynamics in terms of increased interpersonal trust, improved internal communication and information flow, reduced unconstructive judgments between colleagues, and fewer complaints. A more deliberate and thoughtful approach to workplace behaviour and a greater efficiency in complaint resolution processes were observed.

### Identification of contextual factors

The analysis identified four main factors that were decisive in the deployment of the project and the observed benefits observed across the four units studied:

1. The bottom-up co-construction approach, providing various opportunities to stimulate worker participation and the mobilisation of their contributions: context analysis, participatory governance and worker involvement in key decision-making stages within co-construction workshops, frequent opportunities for exchange during implementation, and worker involvement in evaluation;
2. The involvement of team leaders in project design and management: upstream involvement in grant application preparation, leading role in co-construction workshops, involvement in team mobilisation, involvement in steering the implementation of action plans, and time reserved for the project on the management meeting agenda;
3. Investments made by organisations to support front-line workers’ engagement in the project: protected free time for workers involved in project activities, financial rewards to compensate for the extra time required to invest in certain activities, and implementation of follow-up mechanisms;
4. Efforts to promote the project within organisations: discussions at team and management meetings, peer-to-peer project information sharing meetings, dissemination of interim results, and posters.

Three other factors were identified as constraints to the implementation of the project:

1. Staff shortages and high turnover, reducing the teams’ availability to invest in the project. The project even had to be interrupted for a time due to the lack of availability of the staff and other practical constraints related to the COVID-19 pandemic (e.g., telework, difficulty of holding in-person meetings);
2. The additional burden imposed by the project on work teams. The bottom-up approach favoured by the project implied a substantial commitment from workers at every stage, creating a situation of conflict for them between the demands of the project and other organisational priorities;
3. The low level of commitment of some team members to the project. This low level of commitment has been explained by elements linked to the management context: supervisors’ management style, workers’ lack of confidence in the management team, workers’ perception of insufficient support from their immediate supervisors.

## Discussion

The aim of this project was to show how the co-construction process, through collaborative strategies, engages staff in a process of collaboration, transformation, and improvement of their working environment. Faced with the daily challenges of their work environment, the teams were offered opportunities and support through this project to find solutions themselves and implement them. This model, which builds on examples of several healthcare institutions elsewhere in Canada and internationally [29], lays a foundation for sustainable transformation.

### Participatory approach: Added value in oncology

Despite a difficult context for mobilising workers during the implementation of our project (the demanding nature of oncology work being exacerbated by the COVID-19 pandemic), workers, in general, showed enthusiasm for the project, motivated by its perceived relevance and potential benefits. The participatory approach was reflected in a range of activities that provided both workers and local managers opportunities to take part in examining work situations and bringing solutions to identified problems.

Support from an external research team with financial backing from the Ministry of Health and Social Services proved to be a key enabler in advancing this bottom-up initiative, which actively engaged a range of stakeholders, including frontline staff, clinical leaders, and non-clinical contributors. Participation was further facilitated through a combination of supportive measures: protected free time to encourage involvement, financial incentives, oversight by team leaders throughout all project stages, and operational guidance from a dedicated research project coordinator who assumed essential tasks, such as recruiting experts, organising workshops and training sessions, and ensuring follow-up. These conditions helped secure meaningful engagement from both frontline personnel and management. Indeed, as evidenced in the literature: the participatory approach serves as a catalyst for transformation in oncology, empowering healthcare professionals and enhancing teams’ resilience in the face of complex care challenges. This approach increasingly emerges as a critical driver of professional engagement. However, implementing such an approach demands strong institutional commitment, a culture of trust, and structured support systems to ensure genuine and sustained involvement, each of which are principles that guided the design of our project. A meta-analysis of strategies aimed at minimizing physician burnout revealed that physicians could benefit substantially from interventions to reduce burnout, particularly organizational strategies, recognizing that burnout is rooted in issues related to the work environment and organizational culture [30].

Similarly, Lejeune et al. [31] have shown that a participatory approach that builds on interprofessional team meetings, in-service training, team support, and a project-based approach impacts positively on work engagement, job satisfaction, and care quality satisfaction. Beyond technical outcomes, this approach nurtures rich, though often invisible, interpersonal dynamics, and when reinforced by participatory governance, these dynamics facilitate the exchange of best practices, particularly through the influence of informal leaders [32]. It helps to increase the relevance, acceptability, and sustainability of evidence-based practices by aligning them to real-world contexts [33].

### Incremental change and continuous improvement processes

According to respondents, this project did not profoundly disrupt their environment, but it did provide empirical, contextual, and experiential data that allowed them to better address the problems they had identified and prioritised. Our project highlighted the specific challenges of this type of bottom-up co-construction approach, emphasising the need to support such projects over time to achieve long-term sustainability. The results obtained in this work show that the changes made were relatively modest in the short term. However, the direct involvement of workers in the co-construction of improvements in their working conditions and experience led to positive changes, albeit incremental, effects. Indeed, as shown by the examples of similar projects, such as those of Burrill et al. [29], a continuous improvement approach requires an incremental and repeated progression of activities for real change to occur, as well as the commitment of leaders to transform their leadership and focus on a few priorities, as was the case in our project.

In our case, most participants considered the activities implemented as solutions to be relevant while emphasising that they did not always lead to major changes. This observation highlights the need for repetitive cycles of engagement in this type of improvement process. The cultural changes required, and the actions needed to achieve them, are not always natural and quick. Skills needed for the use of problem-solving techniques have to be taught and supported through mentoring and coaching over a longer period until they become part of the daily habits of workers [34, 35]. Although such a project needs a latent period before bearing fruit, it remains essential to establish lasting and positive change. The creation of a common language and the adoption of new behaviours within teams strengthens employee buy-in and prepares the ground for long-term sustainable effects. Maintaining this type of project over time is essential to foster a culture of continuous improvement and engagement, not only for frontline workers but also for organisational leaders. Offering oncology workers opportunities to learn how to effectively face challenges in their work environment has been shown to reduce the prevalence of burnout and psychological distress, frequently reported among them [36–38]. In addition, it benefits the oncology unit and the healthcare facility as a whole while promoting staff longevity and quality of patient care [10].

### Contextual factors and sustainability

To ensure the success and sustainability of this type of project, it is essential to consider certain organisational factors. Active support from managers is necessary to transform leadership within units. Conditions such as funding, staff commitment, and balancing reflection, planning, and action are essential to giving workers room for innovation. Several studies have also highlighted the importance of worker autonomy, visible and supportive leadership, and the development of good relationships between colleagues as crucial elements in preventing burnout and fostering team engagement [3, 39–45].

The bottom-up co-construction approach reflects the model behind the high-involvement work systems that have been demonstrated to improve employee motivation, autonomy, and ownership. It is distinguished by numerous opportunities for frontline staff to participate, ranging from context analysis and participatory governance to active participation in co-construction workshops and evaluation [46]. Furthermore, the early and continuous participation of team leaders in the project’s planning and implementation is consistent with research showing how important distributed leadership is for inspiring teams and sustaining change [47, 48].

The lack of time and resources in high-pressure healthcare settings is another well-documented obstacle to participation that is addressed by the investments made by organisations, such as allocating protected time and providing financial incentives [49]. Maintaining project visibility through peer-to-peer interactions, regular communications, and the sharing of interim results is in line with research that demonstrates how fostering a sense of purpose and facilitating open communication improve collective engagement [50]. Likewise, constraints observed—such as staff shortages, high turnover, the additional workload generated by participatory processes, and the limited commitment of certain team members—echo challenges frequently cited in the literature on participatory interventions in healthcare settings [51, 52]. Indeed, excessive demands run the danger of causing conflicts between project activities and other organisational priorities, while high turnover and a lack of supervisor support can erode motivation and confidence [53].

Taken together, these results highlight how crucial it is to actively address factors that influence the success of participatory change projects while simultaneously managing enablers such as resources, time, and leadership commitment.

### Limits

The primary limitations of this study stem from the highly specific context of each of the four units examined, calling for a certain caution in transferring the findings to other settings. Only a single improvement cycle could be carried out within the four units of the participating institutions, and there was considerable variability among them. Also, participation rates varied significantly across the different units, and there may be a potential selection bias, as participants who chose to respond may already have been more engaged within their respective units. These variations may have influenced the results observed in the present study, while nevertheless allowing for a reflection of diverse organisational contexts. In addition, with the qualitative data, we did not measure a specific outcome; rather, we focused on the changes experienced by workers in their work environment.

Furthermore, the project was implemented in an exceptional context shaped by the COVID-19 pandemic. Nonetheless, these experiments conducted in these units may inspire other units, organisations, researchers, managers, and decision-makers working in oncology and across the healthcare system to undertake similar projects aimed at improving the well-being of workers and their environment. The diverse contexts provided by these units could offer valuable insights. However, variations may exist across geographic regions, highlighting the need for interventions tailored to each regional context. As indicated in the methods section, all the data of this study were cross-referenced, using multiple sources: one-on-one interviews and the collection of documentary data, including survey results. Rigorous monitoring was also carried out at each phase of the project, with thorough documentation and analysis of the associated results. This greatly facilitated the documentary research and its analysis throughout the work of writing this article.

## Conclusion

This study demonstrated how a bottom-up co-construction approach lever to engage workers in improving their work experiences. It highlights the critical importance of engaging frontline workers’ participation in addressing key challenges in the oncology work environment. Although the outcomes were modest in the short term, the incremental improvements observed in these areas provide valuable insights into the potential for sustainable change.

Despite the difficulties faced—such as staff shortages, competing priorities, and the challenges of implementing changes during a global pandemic—the project demonstrated that active worker participation can serve as a powerful catalyst for positive transformation. The solutions implemented relied on a continuum of activities that, while not always immediately effective in reducing burnout, were successful in fostering stronger team dynamics, improving communication, and promoting a greater sense of engagement among workers. Maintaining a continuous improvement approach where small, iterative changes are made over time in oncology care teams to build resilience and enhance well-being is crucial to ensuring the long-term success and sustainability of such initiatives. Ultimately, this study not only contributes to the growing body of research on resilience in healthcare but also presents an intervention that can serve as a practical example for other organisations to adapt when implementing similar initiatives. The knowledge generated provides guidance for transforming practices, and future mixed methods studies could combine quantitative and qualitative data while focussing on different healthcare contexts.

## Supplementary Information

Below is the link to the electronic supplementary material.

Supplementary Material 1

Supplementary Material 2

## Data Availability

The documents mentioned in this article are available, kept by the corresponding author, and can be consulted by sending a request by email.

## Abbreviations

QDA: Qualitative Data Analysis
COVID-19: Coronavirus Disease 2019

## References

[1] Dönmez AA, Ovayolu Ö, Ovayolu N, Yılmaz S, Karayurt Ö, Çürük GN, et al. Quality of work life and working conditions among oncology nurses: A national online descriptive cross-sectional study. Arch Environ Occup Health. 2023;78:131–41. 10.1080/19338244.2022.2063240. 35412450 10.1080/19338244.2022.2063240

[2] Guariglia L, Terrenato I, Iacorossi L, D’Antonio G, Ieraci S, Torelli S, et al. Moral Distress in Oncology: A Descriptive Study of Healthcare Professionals. Int J Environ Res Public Health. 2023;20:5560. 10.3390/ijerph20085560. 37107841 10.3390/ijerph20085560 PMC10139085

[3] Soheili M, Taleghani F, Jokar F, Eghbali-Babadi M, Sharifi M. Oncology Nurses’ Needs Respecting Healthy Work Environment in Iran: A Descriptive Exploratory Study. Asia-Pac J Oncol Nurs. 2021;8:188–96. 10.4103/apjon.apjon_64_20. 33688568 10.4103/apjon.apjon_64_20 PMC7934596

[4] Eche IJ, Phillips CS, Alcindor N, Mazzola E. A Systematic Review and Meta-analytic Evaluation of Moral Distress in Oncology Nursing. Cancer Nurs. 2023;46:128–42. 10.1097/ncc.0000000000001075. 35283474 10.1097/NCC.0000000000001075

[5] Fillion L, Dupuis R, Tremblay I, De Grâce G-R, Breitbart W. Enhancing meaning in palliative care practice: a meaning-centered intervention to promote job satisfaction. Palliat Support Care. 2006;4:333–44. 17133893 10.1017/s1478951506060445

[6] Kelly D, Ross S, Gray B, Smith P. Death, dying and emotional labour: problematic dimensions of the bone marrow transplant nursing role? J Adv Nurs. 2000;32:952–60. 11095235

[7] Hamric AB, Blackhall LJ. Nurse-physician perspectives on the care of dying patients in intensive care units: Collaboration, moral distress, and ethical climate*. Crit Care Med. 2007;35:422. 10.1097/01.CCM.0000254722.50608.2D. 17205001 10.1097/01.CCM.0000254722.50608.2D

[8] Boyle DA. Countering compassion fatigue: a requisite nursing agenda. Online J Issues Nurs. 2011;16. 10.3912/OJIN.Vol16No01Man02 21800933

[9] Lee JS, Akhtar S. Effects of the workplace social context and job content on nurse burnout. Hum Resour Manage. 2011;50:227–45.

[10] Gillman L, Adams J, Kovac R, Kilcullen A, House A, Doyle C. Strategies to promote coping and resilience in oncology and palliative care nurses caring for adult patients with malignancy: a comprehensive systematic review. JBI Database Syst Rev Implement Rep. 2015;13:131–204. 10.11124/jbisrir-2015-1898. 10.11124/jbisrir-2015-1898 26455609

[11] Taylor B, Barling J. Identifying sources and effects of carer fatigue and burnout for mental health nurses: a qualitative approach. Int J Ment Health Nurs. 2004;13:117–25. 15318906 10.1111/j.1445-8330.2004.imntaylorb.doc.x

[12] Burston AS, Tuckett AG. Moral distress in nursing: contributing factors, outcomes and interventions. Nurs Ethics. 2013;20:312–24. 10.1177/0969733012462049. 23275458 10.1177/0969733012462049

[13] Morris SE, Tarquini SJ, Yusufov M, Adolf E, Amonoo HL, Bain PA, et al. Burnout in psychosocial oncology clinicians: A systematic review. Palliat Support Care. 2021;19:223–34. 10.1017/S147895152000084X. 32895081 10.1017/S147895152000084X

[14] Vailati A, Marcomini I, Di Niquilo M, Poliani A, Rosa D, Villa G, et al. Beyond Care: A Scoping Review on the Work Environment of Oncology Nurses. Nurs Rep. 2025;15:324. 10.3390/nursrep15090324. 41003279 10.3390/nursrep15090324 PMC12472513

[15] HaGani N, Yagil D, Cohen M. Burnout among oncologists and oncology nurses: A systematic review and meta-analysis. Health Psychol Off J Div Health Psychol Am Psychol Assoc. 2022;41:53–64. 10.1037/hea0001155. 10.1037/hea0001155 35113585

[16] McCalman J, Bailie R, Bainbridge R, McPhail-Bell K, Percival N, Askew D, et al. Continuous Quality Improvement and Comprehensive Primary Health Care: A Systems Framework to Improve Service Quality and Health Outcomes. Front Public Health. 2018;6:76. 10.3389/fpubh.2018.00076. 29623271 10.3389/fpubh.2018.00076 PMC5874897

[17] Miles M. Continuous Improvement. A 6 Stages to Follow and Why It’s Important. 2022. https://www.betterup.com/blog/continuous-improvement. Accessed 6 Oct 2023.

[18] Bakker D, Fitch MI, Green E, Butler L, Olson K. Oncology nursing: Finding the balance in a changing health care system. Can Oncol Nurs J Rev Can Nurs Oncol. 2006;16:79–98. 10.5737/1181912x1627987. 10.5737/1181912x1627987 17036592

[19] Karasek R, Brisson C, Kawakami N, Houtman I, Bongers P, Amick B. The Job Content Questionnaire (JCQ): an instrument for internationally comparative assessments of psychosocial job characteristics. J Occup Health Psychol. 1998;3:322. 9805280 10.1037//1076-8998.3.4.322

[20] Albrecht SL. Challenge demands, hindrance demands, and psychological need satisfaction. J Pers Psychol. 2015.

[21] Netemeyer RG, Boles JS, McMurrian R. Development and validation of work–family conflict and family–work conflict scales. J Appl Psychol. 1996;81:400–10. 10.1037/0021-9010.81.4.400.

[22] Maslach C, Jackson SE, Leiter MP. Maslach burnout inventory. Scarecrow Education; 1997.

[23] Van der Beek D, Schraagen JM. ADAPTER: Analysing and developing adaptability and performance in teams to enhance resilience. Reliab Eng Syst Saf. 2015;141:33–44.

[24] Patriarca R, Di Gravio G, Costantino F, Falegnami A, Bilotta F. An analytic framework to assess organizational resilience. Saf Health Work. 2018;9:265–76. 30370158 10.1016/j.shaw.2017.10.005 PMC6130002

[25] Temkin-Greener H, Zheng NT, Katz P, Zhao H, Mukamel DB. Measuring work environment and performance in nursing homes. Med Care. 2009;47:482–91. 19330892 10.1097/mlr.0b013e318190cfd3 PMC2663940

[26] Vézina M, Cloutier E, Stock S, Lippel K, Fortin É, Delisle A, et al. Enquête québécoise sur des conditions de travail, d’emploi, et de santé et de sécurité du travail (EQCOTESST). Québec: Institut de recherche Robert-Sauvé en santé et en sécurité du travail; 2011.

[27] Stevens N, Westerhof GJ. Partners and others: Social provisions and loneliness among married Dutch men and women in the second half of life. J Soc Pers Relatsh. 2006;23:921–41. 10.1177/0265407506070474.

[28] Idler EL, Benyamini Y. Self-Rated Health and Mortality: A Review of Twenty-Seven Community Studies. J Health Soc Behav. 1997;38:21–37. 10.2307/2955359. 9097506

[29] Burrill G, Parker J, Fitzgerald E. Creating a culture of excellence - How healthcare leaders can build and sustain continuous improvement. 2019;50.

[30] De Simone S, Vargas M, Servillo G. Organizational strategies to reduce physician burnout: a systematic review and meta-analysis. Aging Clin Exp Res. 2021;33:883–94. 10.1007/s40520-019-01368-3. 31598914 10.1007/s40520-019-01368-3

[31] Lejeune J, Fouquereau E, Chênevert D, Coillot H, Chevalier S, Gillet N, et al. The Participatory Approach: A Specific French Organizational Model at the Department Level to Serve the Quality of Work Life of Healthcare Providers and the Quality of Care in Pediatric Oncology. Cancer Manag Res. 2021;13:2763–71. 10.2147/CMAR.S284439. 33790650 10.2147/CMAR.S284439 PMC8006951

[32] Colladon AF, Grippa F, Broccatelli C, Mauren C, Mckinsey S, Kattan J, et al. Boosting advice and knowledge sharing among healthcare professionals. J Knowl Manag. 2023;27:2017–33. 10.1108/JKM-06-2022-0499.

[33] Ramanadhan S, Davis MM, Armstrong R, Baquero B, Ko LK, Leng JC, et al. Participatory implementation science to increase the impact of evidence-based cancer prevention and control. Cancer Causes Control CCC. 2018;29:363–9. 10.1007/s10552-018-1008-1. 29417296 10.1007/s10552-018-1008-1 PMC5858707

[34] Graban M, Swartz JE. Healthcare kaizen: engaging front-line staff in sustainable continuous improvements. CRC Press. 2018.

[35] Hopper AM. Continuous Improvement Strategies: How to Manage, Motivate, and Retain Staff. Boca Raton: CRC; 2018. 10.4324/9781315154237.

[36] Barrett L, Yates P. Oncology/haematology nurses: a study of job satisfaction, burnout, and intention to leave the specialty. Aust Health Rev. 2002;25:109–21. 12136551 10.1071/ah020109

[37] Grunfeld E, Zitzelsberger L, Coristine M, Whelan TJ, Aspelund F, Evans WK. Job stress and job satisfaction of cancer care workers. Psycho-Oncology J Psychol Soc Behav Dimens Cancer. 2005;14:61–9. 10.1002/pon.820 15386787

[38] Isikhan V, Comez T, Danis MZ. Job stress and coping strategies in health care professionals working with cancer patients. Eur J Oncol Nurs. 2004;8:234–44. 15304231 10.1016/j.ejon.2003.11.004

[39] Abdali Bardeh M, Naji S, Zarea K. The study of job stress and tension management among oncology nurses of Ahvaz Hospitals in 2015. Int J Med Res Health Sci. 2016;5:189–99.

[40] Bährer-Kohler S. Burnout for experts: Prevention in the context of living and working. Springer; 2013.

[41] Bakker D, Strickland J, MacDonald C, Butler L, Fitch M, Olson K, et al. The context of oncology nursing practice: An integrative review. Cancer Nurs. 2013;36:72–88. 23235501 10.1097/NCC.0b013e31824afadf

[42] Barbour LC. Exploring oncology nurses’ grief: A self-study. Asia-Pac J Oncol Nurs. 2016;3:233–40. 27981166 10.4103/2347-5625.189817 PMC5123519

[43] Jones MC, Wells M, Gao C, Cassidy B, Davie J. Work stress and well-being in oncology settings: a multidisciplinary study of health care professionals. Psychooncology. 2013;22:46–53. 10.1002/pon.2055. 21956976 10.1002/pon.2055

[44] Shanafelt T, Dyrbye L. Oncologist burnout: causes, consequences, and responses. J Clin Oncol. 2012;30:1235–41. 22412138 10.1200/JCO.2011.39.7380

[45] Trufelli DC, Bensi CG, Garcia JB, Narahara JL, Abrão MN, Diniz RW, et al. Burnout in cancer professionals: a systematic review and meta-analysis. Eur J Cancer Care (Engl). 2008;17:524–31. 18771533 10.1111/j.1365-2354.2008.00927.x

[46] Boxall P, Macky K. Research and theory on high-performance work systems: progressing the high‐involvement stream. Hum Resour Manag J. 2009;19:3–23.

[47] Chreim S, Williams BE, Hinings CR. Interlevel influences on the reconstruction of professional role identity. Acad Manage J. 2007;50:1515–39.

[48] Denis J-L, Lamothe L, Langley A. The dynamics of collective leadership and strategic change in pluralistic organizations. Acad Manage J. 2001;44:809–37.

[49] Afsar B, Masood M, Umrani WA. The role of job crafting and knowledge sharing on the effect of transformational leadership on innovative work behavior. Pers Rev. 2019;48:1186–208.

[50] Hewison A, Gale N, Yeats R, Shapiro J. An evaluation of staff engagement programmes in four National Health Service Acute Trusts. J Health Organ Manag. 2013;27:85–105. 10.1108/14777261311311816. 23734478 10.1108/14777261311311816

[51] Brunetto Y, Farr-Wharton R, Shacklock K. Supervisor‐nurse relationships, teamwork, role ambiguity and well‐being: Public versus private sector nurses. Asia Pac J Hum Resour. 2011;49:143–64.

[52] Van Bogaert P, Peremans L, Van Heusden D, Verspuy M, Kureckova V, Van de Cruys Z, et al. Predictors of burnout, work engagement and nurse reported job outcomes and quality of care: a mixed method study. BMC Nurs. 2017;16:1–14. 28115912 10.1186/s12912-016-0200-4 PMC5241948

[53] Dixon-Woods M, McNicol S, Martin G. Ten challenges in improving quality in healthcare: lessons from the Health Foundation’s programme evaluations and relevant literature. BMJ Qual Saf. 2012;21:876–84. 22543475 10.1136/bmjqs-2011-000760 PMC3461644

