# Supplementary material for: Enhancing employee experience and workplace well-being in oncology: a bottom-up co-construction approach in healthcare institutions in Québec
Source: BMC Health Serv Res. 2026 Jun 5;26:1103. doi: 10.1186/s12913-026-14881-9 (PMC13471622; doi:10.1186/s12913-026-14881-9)
Supplement: Supplementary file 1 [file 12913_2026_14881_MOESM1_ESM.docx]

## Supplementary material 1: Interview guide
